# Extraction and Physicochemical Characterization of Chitin Derived from the Asian Hornet, *Vespa velutina* Lepeletier 1836 (Hym.: Vespidae)

**DOI:** 10.3390/molecules25020384

**Published:** 2020-01-17

**Authors:** Xesús Feás, M. Pilar Vázquez-Tato, Julio A. Seijas, Anna Pratima G. Nikalje, Francisco Fraga-López

**Affiliations:** 1 Academy of Veterinary Sciences of Galicia, Edificio EGAP, Rúa Madrid, No. 2-4, 15707 Santiago de Compostela, (A Coruña), Spain; 2 Departamento de Química Orgánica, Facultad de Ciencias, Universidad of Santiago De Compostela, Alfonso X el Sabio, 27002 Lugo, Spain; pilar.vazquez.tato@usc.es (M.P.V.-T.); julioa.seijas@usc.es (J.A.S.); 3 Department of Chemistry, Wilson College, Girgaon Chawpatty, Mumbai 400007, Maharashtra, India; annapratimanikalje@gmail.com; 4 Departamento de Física Aplicada Departamento de Física Aplicada, Facultad de Ciencias, Universidad de Santiago de Compostela, Avda. Alfonso X El Sabio s/n, 27002 Lugo, Spain

**Keywords:** chitin, *Vespa velutina*, Asian hornet, polymer, invasive species, insects

## Abstract

Fifteen years ago, at least one multimated female yellow-legged Asian hornet (*Vespa velutina* Lepeletier 1836) arrived in France, which gave rise to a pan-European invasion. In this study, the isolation and characterization of chitin (CHI) that was obtained from *Vespa velutina* (CHI_VV_) is described. In addition, an easy procedure is carried out to capture the raw insect, selectively and with high rates of success. The chitin contents of dry VV was observed to be 11.7%. Fourier transform infrared spectroscopy (FTIR), solid-state NMR (ssNMR), elemental analysis (EA), scanning electron microscopy (SEM), and thermogravimetric analysis (TG) characterized the physicochemical properties of CHI_VV_. The obtained CHI_VV_ is close to pure (43.47% C, 6.94% H, and 6.85% N), and full acetylated with a value of 95.44%. Additionally, lifetime and kinetic parameters such as activation E and the frequency factor A using model-free and model-fitting methods, were determined. For CHI_VV_ the solid state mechanism that follows the thermodegradation is of type F2 (random nucleation around two nuclei). The invasive Asian hornet is a promising alternative source of CHI, based on certain factors, such as the current and probable continued abundance of the quantity and quality of the product obtained.

## 1. Introduction

The chitin (CHI) is the most abundant biopolymer in nature, behind only cellulose. Structurally, it is the simplest of the glycosaminoglycans, being a β (1→4) linked linear homopolymer of *N*-acetylglucosamine (GlcNAc, [C_8_H_13_O_5_N] n, where n››1). It primarily exists in alga [1,2], fungi [3], arthropods (crab, shrimp, crayfish, and insects) [4], copepod, and mollusks (squid) [5]. It was cited that approximately 100 billion tons of CHI are produced, as a major structural component in the exoesqueleton from the above organisms [6], being around 80,000 t obtained per year from the marine by-products by industry for commercial exploitation. [7].

CHI is usually converted into chitosan and partially acetylated chitosan oligomers (COS) by hydrolysis of the acetamide groups due to its poor solubility in most organic solvents as well as water. A soluble material can be obtained in aqueous acid medium, with three attractive reactive sites enabling modification, including one primary amine and two primary or secondary hydroxyl groups per glucosidic unit [8].

This polycationic polymer can be processed into several forms, and chemically or enzymatically modified, with a definite chemical composition and ordered structure [9]. Definitively, they have emerged as a new class of physiological materials of highly sophisticated functions, being referred the CHI as the “undisputed biomolecule of great potential” [10].

The great variety of applications of chitosan in the field of biomaterials is due to its excellent properties when interacting with the human body: bioactivity [11], antimicrobial and antifungal activity [12,13], immunostimulation [14], chemotactic action, enzymatic biodegradability, mucoadhesion, and epithelial permeability [15], which supports the adhesion and proliferation of different cell types [16]. With the above properties, CHI derivatives serve a broad range of applications and these materials have advanced at a dramatic pace into many fields, including skin, wound, and burn management [17]; drug delivery and pharmaceutical applications [18]; tissue engineering [19]; dentistry [20]; plant science and agriculture [21]; veterinary science [22]; cosmetics and cosmeceutical [23]; food and nutraceuticals [24]; and, paper industry [25], among others.

Despite the widespread occurrence of CHI, until now the main commercial sources have been crab, prawn, lobster, krill, and shrimp shells. The resulting CHI needs to be graded in terms of purity and colour, since residual protein and pigment can cause problems for further utilization, especially for biomedical products [26].

Two new sources as fungi and insect species have been recently researched as an alternative origin for these functional materials in order to expand the chitin-chitosan based source and obtain a better material and more consistent quality [3,7,27,28,29,30,31]. The insect and fungal biopolymers have many features that can make them more advantageous than those biopolymers from seafood waste origin [32], due to potential advantages in terms of homogenous polymer length, high degree of deacetylation and solubility over the current marine source [7]. The needs in new sources are very acute since the traditional methods of CHI by catches of shellfish and complex selection procedures of desired product are largely limited. Use of insects for CHI manufacture is grounded by economic benefit, being much cheaper and easier than by the treating of crustaceans or mushrooms [33].

The yellow-legged Asian hornet (*Vespa velutina* Lepeletier 1836 (Hymenoptera: Vespidae)) is naturally distributed in Southeast Asia, India, and China; however, fifteen years ago, at least one multimated female yellow-legged Asian hornet arrived in France. The species has subsequently expanded its range and it is now presently recognized as a pan-European threat after being detected in Spain (2010), Portugal (2011), Belgium (2011), Italy (2012), Germany (2014), and the Netherlands (2018), as well as on islands such as Majorca in the Balearic Islands (2015), England, and the Channel Islands (2016) [34]. The large numbers of *Vespa velutina* that were captured for the control of this specie can provide an abundant and stable source for the production of CHI [35,36].

In the present work, physicochemical properties of the chitin isolated from *Vespa velutina* were determined for the first time. First, a procedure is presented to selectively catch the invasive insect. Secondly, the CHI was purified while using alkaline and acid treatments, followed by decolourization and, finally, the obtained *Vespa velutina* (VV) chitin were characterized by Fourier transform infrared spectroscopy (FTIR), elemental analysis (EA), scanning electron microscopy (SEM), and thermogravimetric analysis (TGA). Additionally, kinetic parameters such as activation E and the frequency factor A using model-free methods given by Kissinger, Flynn Wall, and Ozawa (FWO), Coats Redfern, were determined. The results that were obtained in the present work allow for us to consider the invasive species *Vespa velutina* as a large-scale and perspective source of chitin/chitosan obtaining.

## 2. Results and Discussion

### 2.1. Insect Source and Chitin Yield 

Approximately 2,289 insects with a total weight of 824.8 g were caught and dried to constant weight. A total of 235.9 g, or 28.6 per cent of the weight of the fresh material, of dry insect bodies were obtained. Therefore, the percent moisture loss of the insects, based on percent of the original weight, was 71.4%. 

The Asian hornet is an insect hunter, specializing in honeybees. Unlike in its area of origin, where the Asian honeybee (*Apis cerana*) has developed defence mechanisms against its attacks, the European honeybee (*Apis mellifera*) does not show effective defence behaviour. The hives are brutally attacked by the Asian hornet coinciding with the development peak of its colony, and the need for more protein to feed its larvae. From this point of view, apiaries are a suitable and selective site, as the obtained results show, to obtain the raw insects. The traps placed show exclusively captures of VV and not of other not-target insects. The results are a reflection of VV activity and the relative abundance of the species present at the time that the traps were allocated. 

The chitin content of VV was recorded to be 11.7% on a dry basis. Dry insects from previous studies have been determined to have CHI in the range of 10–36%, and these organisms have been reviewed and suggested to be used as alternative sources for CHI production [9]. The CHI content of the shells of commercially used organisms, such as crayfish, shrimp, and crap, was referred to be around 20% [28].

### 2.2. Spectroscopic Chitin Analysis

**Figure 1:**
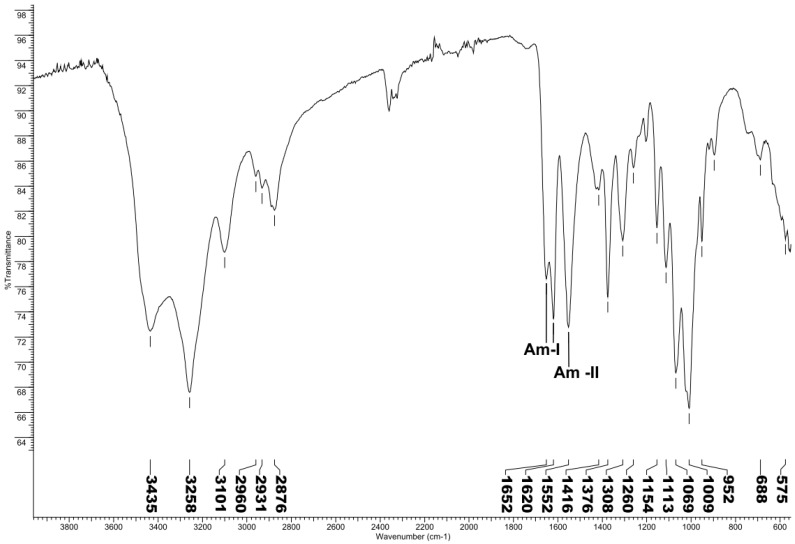
Fourier-transform infrared spectroscopy (FT-IR) spectrum data of chitin extracted from *Vespa velutina*. 1652 and 1620 amide I (Am-I), 1552 amide II (Am-II) characteristic of α-chitin.

Three different allomorphic forms of chitin, as α, β, and γ are known from the literature. FT-IR spectroscopy is one of the fastest and simplest techniques used to determine allomorphic form of chitin. The amide I band of the α form gives two sharp bands at 1652 and 1620 cm^−1^, whereas the β form only presents a single band around 1640 cm^−1^ due to the hydrogen bonds between the molecules [37], as known from many reported studies regarding FT-IR spectrum (Figure 1) of α- and β-chitin.

**Figure 2:**
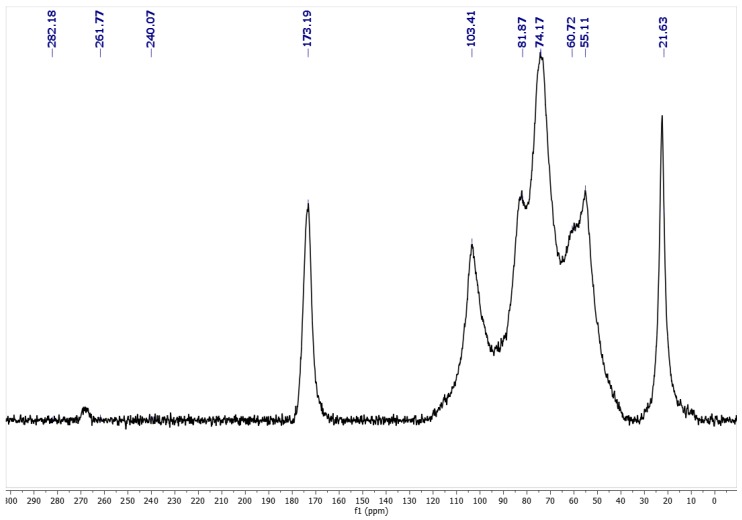
Solid-state CP–MAS ^13^C NMR of chitin extracted from *Vespa velutina*. ^13^C NMR (126 MHz): δ 267.96, 173.17, 104.12, 82.74, 75.34, 60.34, 55.94, 22.79.

Solid-state CP–MAS ^13^C NMR is known to be very sensitive to changes in the local order structure. Solid NMR experiments combine cross-polarization for the sensitivity enhancement of the signal-to-noise ratio with dipolar decoupling for the removal of dipolar interactions from protons and magic-angle spinning for high resolution of chemical shifts. From NMR spectra (Figure 2), α-chitin and β-chitin are easily distinguished. Two signals around 75 and 82 ppm have been assigned to C3, and the C5 carbon atoms in α-chitin are sharply resolved; the signals for β-chitin appear as a singlet [38].

### 2.3. Elemental analysis (EA) and Degree of Acetylation (DA)

Table 1 shows the results of the elemental composition measurements and the theoretical values for CHI_VV_, including carbon, nitrogen, hydrogen, as well as %CHN contents and C/N ratio. Acetylation degree (DA) of CHI_VV_ was calculated while using the elemental analysis results. CHN analysis provides a quick and inexpensive method to check chitin purity, and in conjunction with other techniques, allows for the characterisation of the biopolymer obtained. The chemical composition of polymers is connected with the chemical, physical, and mechanical properties. 

Elemental analysis revealed that CHI_VV_ consisted of 43.47% C, 6.94% H, and 6.85% N, totalling % CHN of 57.26. Experimental C/N for CHI_VV_ was 6.35, being the theoretical value expected 6.86. The DA was calculated to be 95.44%. The results showed that C, N and % CHN contents were found minor that theoretical values. Only experimental H for VV chitin was found to be higher than the theoretical value expected 6.40%. Based on the above results, CHI_VV_ is close to pure and full acetylated. In previous researches using related insects, the % of C, H, and N content in CHI were recorded as 46.62, 6.42, and 6.85 for Vespa crabro; 46.01, 6.71, and 6.34 for Vespa orientalis [28], and 45.5, 6.3, and 6.49 for Vespa crabro, respectively [31].

The determination of the C, H, and N content of CHI is highly recommended for the control quality of the polymer that was obtained after purification from raw material. Moreover, these parameters can give us more information regarding the chemical process used to purificate CHI from a matrix. For example, N in insects mostly occurs in protein and CHI, which combine in different proportions to produce the insect exoskeleton or cuticule. The N content is also a measure of the protein amount that is still present in CHI, not being eliminated in the chemical extraction process [39]. Chitin isolates typically have N content about 7% [40]. DA values that are higher than 100% indicate that some minerals are still present in the CHI structure, not being fully eliminated in the demineralisation step.

### 2.4. Scanning Electron Microscopy (SEM)

**Figure 3:**
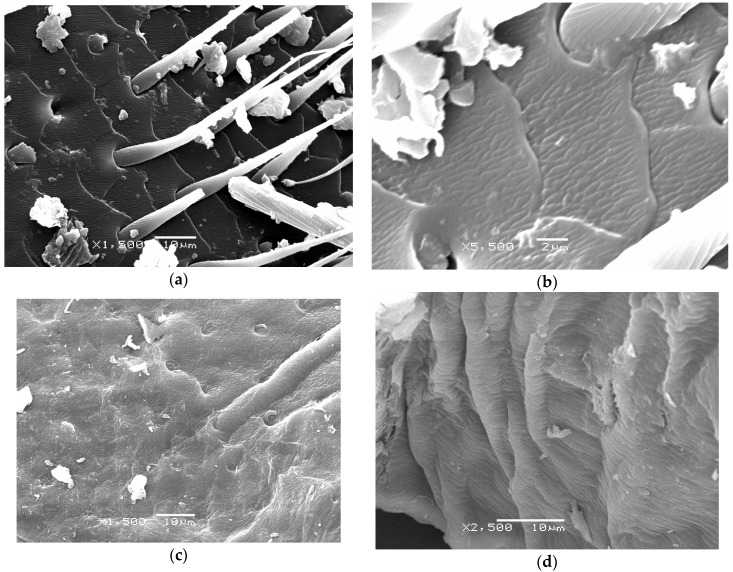
Scanning electron microscopy (SEM) pictures of chitin isolated from *Vespa velutina*. (**a**) magnification ×1500 (scale bar = 10 μm); (**b**) magnification ×5500 (scale bar = 2 μm); (**c**) magnification ×1500 (scale bar = 10 μm) and (**d**) magnification ×2500 (scale bar = 10 μm).

Scanning electron microscopy is a highly suitable method for observing the structure of polymers, in general [41,42,43], and anatomical variation in CHI organization in insect cuticles, in particular [44]. Figure 3a–d shows the diverse nano and micro scale organization of the CHI_VV_.

Morphologies that were identified using SEM and classificated as (1) microporous smooth surface, (2) fish scale shaped nano fibrous surface, (3) adherent fibres with nano pores, and (4) separated fibres with nano pores were reported previously for Vespa crabro [31]. Similar structures were found in the present work for CHI_VV_.

### 2.5. Thermogravimetric Analysis (TG)

Thermogravimetric analysis is the most commonly used technique for characterizing the decomposition and thermal stability of materials under certain conditions. In addition, kinetic parameters can be extracted from thermogravimetric data and predicted half-life [45,46]. The main advantage lies in the simplicity and sensitivity of the technique and in the extensive information that was obtained from a thermogram. The experiments were performed at different heating rates (β) between 5 and 25 °C/min. with increments of 10 °C/min., since a priori, with a low heating rate, the evolution of thermal degradation occurs with greater independence of the measurement method, without necessarily being affected by the test.

**Figure 4:**
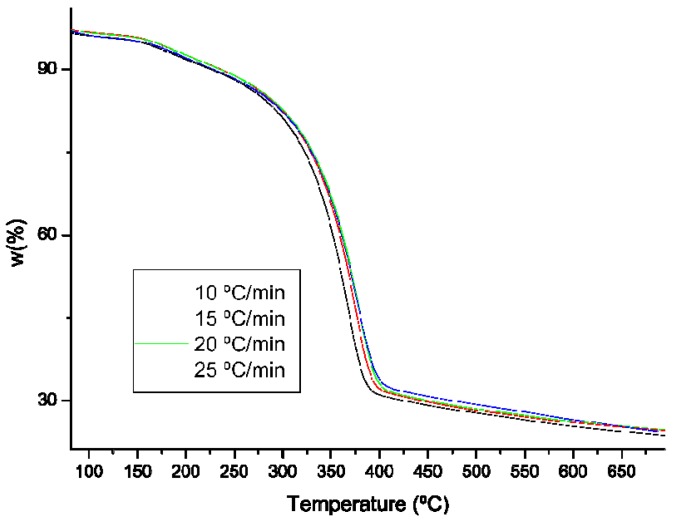
Experimental thermogravimetric (TG) curves at different heating rates of chitin isolated from *Vespa velutina*.

In dynamic tests, mass loss is carried out according to a controlled temperature program. The dynamic methods are divided into differential or integral methods, as they are based on the general velocity equation or its integrated form, respectively. Figure 4 shows the thermodegradation of CHI_VV_ at different heating rates.

The curves have a single-type thermodegradation in a single stage. The inflection points of the curves are taken as a characteristic point for thermodegradation and for the analysis of the different degradation methods. We can observe that, for CHI_VV_, this point occurs at the temperature of 367 °C. Around 700 °C, there is only a 20% residue left. Previous research, where GlcNAc was pyrolized at 200 °C for 30 min., identified pyrazines, pyridines, pyrroles, and furans as thermal degradation products, ranking compound 3-acetamido-5-acetylfuran first [47].

According with the equation calculation process (see Equation (14) in the Material and Methods Section) if ln (*β*/*T_m_*^2^) is represented against the inverse of the temperature, the activation energy can be obtained from the slope of the line. Table 2 shows the values that were used in the calculation of the activation energy for CHI_VV_. The activation energy obtained is 128.6 kJ/mol.

In this way, log *β* can be represented against the inverse of the absolute temperature and obtain the activation energy (*E*) as the slope of the linear adjustment. Table 3 shows the activation energy values that were obtained for CHI_VV_ with different degrees of degradation (α = 0.20, 0.25, 0.30, and 0.35).

The average value of the activation energy is 114.1 kJ/mol for chitin, being this value very close to those that were obtained by the Kissinger method.

According with the equation calculation process (see Equations (27) and (28) in the Material and Methods Section), for each of the solid state reaction mechanisms, one can define a g (α), at each of the heating rates, which will be used for conversions between 0.2 and 0.35. Once the functions g (α) are known, we can make the linear adjustment to obtain the activation energies and their correlation coefficients at each heating rate (Table 4).

From these data and, when comparing with the activation energy obtained by the Kissinger and Flynn_Wall-Ozawa methods, we can highlight that, for CHI_VV_, the solid state mechanism that follows the thermodegradation is of type F2 (random nucleation around two nuclei).

### 2.6. Prediction of the Average Lifetime Considering the Reaction Mechanism

The philosophy of the prediction of the average lifetime is based on identifying the critical reaction that limits the life of a material, studying the kinetics of the said reaction at high temperatures, where the reaction is faster. Finally, the expressions of the kinetic properties are used and the kinetics are extrapolated to much longer reaction times at much lower temperatures, at which the sample will be in service. Obviously, we can also find the shortest life times by extrapolating the kinetics at higher temperatures.

The half-life can be determined once the reaction mechanism is known, without using the expression of *g* (α) of the corresponding solid-state mechanism, from the activation energy and the pre-exponential factor that were calculated from the Flynn-Wall-Ozawa method. The velocity constant can be deduced from the Arrhenius equation as a function of temperature. The study of the thermodegradation kinetics of CHI_VV_ confirms a mechanism F2. The half-life at different temperatures for CHI_VV_ can be observed in Table 5.

The prediction of the average lifetime is a technique that is usually used in the industry to know the probable behaviour of new materials over time [48]. The obtained results show that CHI_VV_ should not be used at temperatures above 60 °C, since its half-life would be only one year. In studies such as those we present at work, it is accepted that thermogravimetric analysis, with its kinetic parameters, is sufficient for determining the lifetime of the material. This time is considered when 5% of the initial mass [49] has been lost or when 5% of the conversion [50] has been reached in a thermogravimetric experiment.

## 3. Materials and Methods 

### 3.1. Sampling of Insects

**Figure 5:**
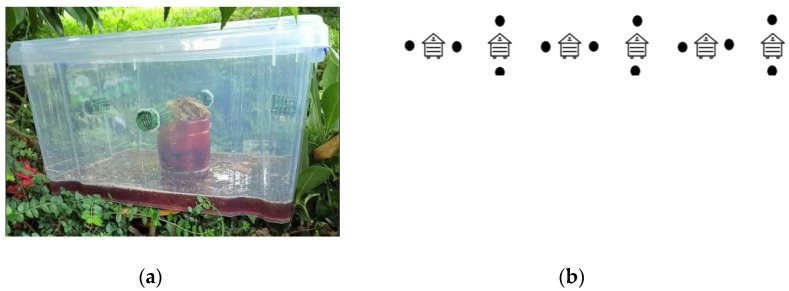
(**a**) The trap used to catch the *Vespa velutina*; (**b**) The (black dots) show the distribution of the traps in the apiary.

During September 2017, adult *Vespa velutina* specimens were obtained while using a trapping method at an apiary in Viveiro (Galicia, Spain), as shown in Figure 5. The beehives (n = 6) had a frontal protective module, as a grid, which prevents the entry of the Asian hornet into the hive. The traps (n = 12) used for capturing the insects consisted of a 15 litre plastic box with a clip on lid and carry handle (volume = 15 l; width = 36.5 cm; depth = 28.5 cm; height = 18.5 cm). A total of four holes were made on the sides of the container for insects to enter. In each hole, a 5 cm snap on roller was inserted with 1 cm of the body outside the box. The attractant used consisted of half blueberry juice, half dark brown bee, r and wax obtained from honeybee combs. After one week in the field, the content inside the traps were removed, and the obtained insects were sent to the laboratory.

### 3.2. Insects Pre-treatment

The insects were washed under the flow of tap water using a metal strainer to remove any remaining matter from the attractant in the trap and paper dried. The number of captured individuals were counted and weighed, obtaining the insect total number (ITN) and insect wet weight (IWW). Afterwards, the insects were oven dried for two days at 70 °C, weighed, and insect dry weight (IDW) was recorded. The difference between IWW and IDW was used to determine the percent moisture of the sample, based on percent of the original weight, as follows:

Moisture (wet basis) = [IWW − IDW) IWW] × 100(1)

Subsequently, oven dried insects were milled using a GRINDOMIX GM 200 (Retsch GmbH, Haan, Germany) and stored in airtight containers in a desiccator.

### 3.3. Extraction of Chitin

Chitin extraction was carried out while using the conventional chemical method that was reported by Kaya et al. [28,31], with modifications. In brief, this involved three major steps, i.e., demineralization, deproteination, and decolourization, carried out as follows.

#### 3.3.1. Demineralisation

This step involved the removal of mineral matter from the insects. The sample (5 g) was treated in 1 M HCl solution (100 mL) at 50 °C for 3 h while using a heating magnetic stirrer (Heildoph, Schawabach, Germany). After this process, the samples were washed with distilled water to remove residual HCl. This was repeated until neutral pH was reached in wash supernatant.

#### 3.3.2. Deproteinization

For deproteinization, the demineralizated samples were treated in 1 M NaOH solution (100 mL) at 60 °C for 8 h. After this process, the samples were washed with distilled water to remove residual HCl. This was repeated until the neutral pH was reached in wash supernatant.

#### 3.3.3. Decolourization

For the removal of the pigments, the demineralizated and deproteinizated samples were mixed with 100 mL 1% sodium hypochlorite solution, at room temperature. The mixture was agitated with a magnetic stirrer for 1 h to decolourize it. Following the filtration process, the samples were thoroughly washed in distilled water before being placed in an oven at 50 °C for one week. The finally obtained chitin was weighed (CHIw) and then stored in desiccator, until further analysis.

### 3.4. Chitin Obtained

The percentage yield of the CHI obtained was calculated as follow:

Yield of chitin = (CHIw − IDW) × 100(2)

### 3.5. Chitin Spectroscopic Characterisation

Fourier-transform infrared spectroscopy (FTIR) was performed while using a Nicolet™ iS10 spectrometer (Thermo Scientific, Madison, USA) that was equipped with a KBr beam splitter and fitted with an attenuated total reflection (ATR) device (Golden Gate, 45 ° single-bounce diamond anvil, Specac, UK). Optical of the spectrometer and ATR device were both purged while using dry air. The samples for FTIR analysis were prepared by grinding the chitin with powdered KBr, in the ratio of 1:5 (Sample: KBr). The samples were scanned at room temperature (25 ± 1 °C) and spectral data between 550 and 4000 cm^−1^ was obtained by collecting 32 scans at 4 cm^−1^ resolution. Corrections for background absorbance and sampling depth of the ATR method were applied.

### 3.6. Solid-state NMR (ssNMR) Analysis

Solid-state CP–MAS 13C NMR experiment was performed using a spectrometer Varian VNMRS-500-WB (Agilent Technologies, Palo Alto, CA, USA) at 126 MHz, while using adamantine as calibration sample.

### 3.7. Chitin Thermogravimetric Analysis (TGA)

Measurements of thermogravimetric analysis (TGA) were performed on a TGA/DSC 1 START_e_ SYSTEM (Metter-Toledo AG, Schwerzenbach, Switzerland), while using alumina crucibles under a stream of N_2._ The system was operated in the dynamic mode in the temperature range 100–900 °C, at different heating rates: 10, 15, 20, and 25 °C. The sample size was approx. 5 mg. The results were processed using the Mettler STARe 9.01 software (Metter-Toledo AG, Schwerzenbach, Switzerland).

#### 3.7.1. Kinetic Methods

Most of the methods of kinetic analysis are based on the hypothesis that, from a simple thermogram, important parameters, such as activation energy, pre-exponential factor, and reaction order, are obtained. Decomposition is one of the phenomena that can occur when a sample undergoes a heating process. A kinetic study is necessary in order to determine the kinetic parameters that characterize this change [51]. For this, the conversion α is previously defined as:

(3) $$\alpha =\frac{m_{i}-m}{m_{i}-m_{f}}$$

m_i_ and m_f_ are, respectively, the initial and final mass at a given instant.

The changes in sample mass can be evaluated as a function of temperature (dynamic method) or as a function of time at constant temperature (isothermal method). In dynamic methods, the temperature increases, generally linearly, according to a heating program.

We can conclude that thermogravimetric analysis, in either of its two methods (dynamic or isothermal), relates conversion, time, and temperature since we have defined conversion based on mass loss, and, consequently, a model could be “designed” Kinetic to describe the thermal degradation of the system. The mathematical model that has been taken into account expresses the reaction rate of the solid state as:

(4) $$\frac{d\alpha }{dt}=k(T)\cdot f(\alpha )$$

Being α the degree of conversion, k (T) the velocity constant, and f (α) the dependence function of the decomposition mechanism.

Since the change in mass is a function of temperature, its effect is introduced through the Arrhenius equation:

(5) $$k(T)=A\cdot e^{-\frac{E}{RT}}$$

where A is the pre-exponential factor, E is the activation energy, and R is the gas constant.

In this type of study, the use of the Arrhenius equation is questioned, although it should be noted that parameters A and E are determined experimentally, and its theoretical interpretation is very difficult [52]. By combining these two equations, the reaction velocity equation can be written in the form:

(6) $$\frac{d\alpha }{dt}=A\cdot e^{-\frac{E}{RT}}\cdot f(\alpha )$$

It should be noted that the velocity also depends on the partial pressure of the gases originating in the thermodegradation (product gases), so this expression should also include a pressure function [52]. However, in most cases, a constant inert gas flow is operated at constant pressure, so that the influence of the product gases can be neglected. If the temperature of the sample constantly changes, that is, if we are working in a dynamic regime, the variation in the degree of conversion can be analysed as a function of the temperature, which, in turn, is dependent on the heating time. In these cases the speed equation is written, as follows:

(7) $$\frac{d\alpha }{dt}=\frac{d\alpha }{dT}\cdot \frac{dT}{dt}=\beta \cdot \frac{d\alpha }{dT}$$

In which α = *dT*/*dt* is defined as the heating rate.

If we clear *dα*/*dT*:

(8) $$\frac{d\alpha }{dT}=\frac{1}{\beta }\cdot \frac{d\alpha }{dt}$$

The combination of expressions (6) and (9) leads to:

(9) $$\frac{d\alpha }{dT}=\frac{1}{\beta }\cdot A\cdot e^{-\frac{E}{RT}}\cdot f(\alpha )$$

Separating variables and integrating this equation from an initial temperature T_0_, which corresponds to a conversion α_0_, to a temperature Tp with a conversion αp we obtain an expression:

(10) $$\int_{\alpha_{0}}^{\alpha }\frac{d\alpha }{f(\alpha )}=\frac{A}{\beta }\int_{0}^{T}e^{-\frac{E}{RT}}dT$$

For small initial temperatures, it is reasonable to assume that *α*_0_ = 0 and admitting that there is no reaction between 0 and *T*_0_ [53], the integral conversion function, g (α), can be defined in this way:

(11) $$g(\alpha )=\int_{0}^{\alpha_{p}}\frac{d\alpha }{f(\alpha )}=\frac{A}{\beta }\int_{0}^{T}e^{-\frac{E}{RT}}dT$$

where A, E, and g (α) or f (α) must be experimentally determined.

In a high number of chemical compounds, the degradation process behaves as a sigmoidal or decelerative function. The expressions of *g* (α) for the different solid-state mechanisms are successfully used in the estimation of reaction mechanisms from dynamic curves [52,54].

#### 3.7.2. Differential Method

The analysis of mass changes, which is produced as a result of varying the heating rate, is the basis of the most powerful differential methods for the study and determination of kinetic parameters. One of the most used is the Kissinger method [55], which uses the inflection point of the thermogram to determine the activation energy.

Kissinger differentiated the general speed equation and obtained:

(12) $$\frac{d^{2}\alpha }{dt^{2}}=\frac{E\beta }{RT^{2}}\cdot \frac{d\alpha }{dt}+A\cdot e^{-\frac{E}{RT}}\cdot f^{\prime }(\alpha )\cdot \frac{d\alpha }{dt}=\left[\frac{E\beta }{RT^{2}}+A\cdot e^{-\frac{E}{RT}}\cdot f^{\prime }(\alpha )\right]\frac{d\alpha }{dt}$$

Particularizing the above equation for the point of inflection at the temperature *T_m_*, at which the maximum degradation occurs:

(13) $$0=\frac{E\beta }{RT_{m}^{2}}+A\cdot e^{-\frac{E}{RT_{m}}}\cdot f^{\prime }(\alpha_{m})$$

Rearranging and taking logarithms, the equation takes the form:

(14) $$\ln\left(\frac{\beta }{T_{m}^{2}}\right)=-\frac{E}{RT_{m}}+\ln\left[f^{\prime }(\alpha_{m})\cdot \frac{AR}{E}\right]$$

#### 3.7.3. Integral Methods

##### Flynn-Wall-Ozawa Method

In cases where the sample is heated at a constant speed [56,57], it is verified that:

(15) $$\frac{d\alpha }{dt}=\frac{d\alpha }{dT}\cdot \frac{dT}{dt}=\beta \cdot \frac{d\alpha }{dT}$$

As already described in the previous section, equation 11 reached a series of considerations. The resolution of this integral is greatly simplified by entering the variable x defined as:

(16) $$x=\frac{E}{RT}$$

Clearing T and differentiating,

(17) $$T=\frac{E}{Rx}$$

(18) $$dT=-\frac{E}{Rx^{2}}dx$$

As we introduce a new variable, we must also change the limits of integration:

(19) $$\begin{array}{l} T\to 0\Rightarrow x\to \infty \\ T\to T\Rightarrow x\to \frac{E}{RT} \end{array}$$

To be able to rewrite the integral, as follows:

(20) $$g(\alpha )=\int_{\infty }^{x}\frac{A}{\beta }\cdot \left(-\frac{E}{R}\right)\cdot \frac{e^{-x}}{x^{2}}\cdot dx=\frac{AE}{\beta R}\cdot p(x)$$

where $p(x)=\int_{\infty }^{0}\frac{e^{-x}}{x^{2}}dx$. This new integral can be evaluated for several values of x and in the literature there are several approaches [58,59]. The Schlömlich expansion is one of the most commonly used series for the estimation of p (x), which was used with good results by Doyle.

(21) $$p(x)=\frac{e^{-x}}{(1+x)x}=\left(1-\frac{1}{x+2}+\frac{2}{\left(x+2)(x+3\right)}-\frac{3}{\left(x+2)(x+3)(x+4\right)}+\ldots \right)$$

Doyle considers it to be sufficient to take only the first two terms of each series. With what:

(22) $$p(x)=\frac{e^{-x}}{x(x+2)}$$

Finding that for x > 20 and taking logarithms could express p (x) as:

(23) $$\log_{10}p(x)\approx -2.315-0.457x$$

Flynn and Wall, on the one hand, and Ozawa on the other, developed a method in which they used the Doyle approach [60] to, without knowing the reaction order, be able to determine the activation energy. According to this, equation 18 is transformed into:

(24) $$g(\alpha )=\frac{AE}{\beta R}\cdot \frac{e^{-x}}{x(x+2)}$$

If we take logarithms and reorder:

(25) $$\log\beta =\log\frac{AE}{Rg(\alpha )}-2.315-\frac{0.457E}{RT}$$

##### Coats–Redfern Method

To solve the temperature integral, Coats and Redfern [61] suggest an asymptotic expansion, so that the integral equation is transformed into:

(26) $$g(\alpha )=\frac{ART^{2}}{\beta E}\left(1-\frac{2RT}{E}\right)e^{-\frac{E}{RT}}$$

Taking logarithms:

(27) $$\ln g(\alpha )=\ln\frac{AR}{\beta E}+2\ln T+\ln\left(1-\frac{2RT}{E}\right)-\frac{E}{RT}$$

Assuming that ln (1-2RT/E) tends to zero for the working conditions used (x > 20), we can write:

(28) $$\ln\frac{g(\alpha )}{T^{2}}=\ln\frac{AR}{\beta E}-\frac{E}{RT}$$

#### 3.7.4. Prediction of the Average Lifetime Considering the Reaction Mechanism

Taking that the half-life is defined as the time necessary to reach 5% of the conversion into account, the equation for determining the half-life once the reaction mechanism is known is as follows, introducing in g (α) the solid state equations corresponding to CHI_VV_:

(29) $$t=\frac{g(0.05)}{k}$$

### 3.8. Elemental Analysis

The total carbon, nitrogen, and hydrogen in CHI were estimated while using C H N elemental analyser (Flash EA 1112 Series, Thermo Finnigan, Italy), which consisted of the system unit, a MAS 200 autosampler for solid samples, and a Windows compatible computer with Eager 300 software. The equipment was calibrated and standardized while using BBOT Standard {*2, 5-bis (5-tert-butyl-benzoxazol-2-yl)-thiopen*, C_26_H_26_N_2_O_2_S, Thermo Finnigan, Italy)}. The minimum detection limit for C, N, and H was calculated to be 0.08%.

### 3.9. Degree of Acetylation (DA)

The acetylation degree (DA) of the chitin that was obtained from Asian hornet samples was calculated according to [62] while using the elemental analysis results

DA = [(C/N − 5.145)/1.72] × 100(30)

### 3.10. Scanning Eectron Microscopy (SEM)

The morphology of chitin obtained was observed while using SEM (JEOL JSM-6360LV, Tokyo, Japan). The samples required a gold ion coating. First, a sample was mounted on metallic studs with double-sided conductive tape. Gold ion coating was then applied with a sputter coater (Bal-tec SCD 050, GmbH, Witten, Germany) for 60 s under vacuum at a current intensity of 60 mA. The acceleration voltage during SEM scanning was 20 kV.

## 4. Conclusions

To the best of our knowledge, this is the first time that the isolation and characterization of chitin that was obtained from *Vespa velutina* is described. The dry weight chitin content of *V. velutina* was 11.7%. The surfaces structures of the Asian hornet chitin samples showed different morphology that mainly consisted of nanofibers and nanopores. Elemental analysis revealed that chitin from *V. velutina* consisted of 43.47% C, 6.94% H, and 6.85% N. The acetylation degree value was calculated as 95.44%. The study of the thermodegradation kinetics of *V. velutina* chitin confirms a mechanism F2 (random nucleation around two nuclei). The Asian hornet is a promising alternative source of chitin based on certain factors, such as the current and probable continued abundance of this invasive species and the trapping procedure proposed and employed, as well as the quality of the product obtained. 

## Figures and Tables

**Table 1:** Elemental composition measurements and theoretical values for chitin from *Vespa velutina*.

	% C	% H	% N	% CHN	C/N	DA%
*Vespa velutina* chitin	43.47	6.94	6.85	57.26	6.35	95.44
Theoretical value of chitin	47.29	6.40	6.90	60.59	6.86	100
Found-Theoretical	−3.82	0.54	−0.05	−3.33	−0.51	−4.56

**Table 2:** Inflection point temperatures at different rates for chitin.

B (°C/min)	T (°C)
5	363
10	367.8
15	369.3
20	376.3

**Table 3:** Degradation energies at different conversions for chitin obtained from *Vespa velutina*.

α	E_a_(kJ/mol)
5	117.8
10	118.9
15	114.1
20	105.5

**Table 4:** Degradation energies at different heating rates and different thermodegradation mechanisms for *Vespa velutina* chitin.

Solid State Process	Mechanism	5 °C/min	10 °C/min	15 °C/min
Nucleation and growth (Avrami Equation (1))	A_2_	35.2	53.6	44.7
Nucleation and growth (Avrami Equation (2))	A_3_	16.9	33.8	11.3
Nucleation and growth (Avrami Equation (3))	A_4_	6.5	17.4	5.9
Phase boundary controlled reaction (one-dimensional movement)	R_1_/F_0_	67.1	53.7	73.6
Phase boundary controlled reaction (contracting area)	R_2_	77.2	62.8	82.7
Phase boundary controlled reaction (contracting volume)	R_3_	80.8	66.07	85.9
One-dimensional diffusion	D_1_	187.7	161.8	181.5
Two-dimensional diffusion	D_2_	200.9	173.7	193.4
Three-dimensional diffusion (Jander eq.)	D_3_	215	186.6	202.4
Three-dimensional diffusion (Ginstling-Brounshtein eq.)	D_4_	205.6	177.9	197.6
Random nucleation with one nucleus on the individual particle	F_1_	88	72.5	92.36
Random nucleation with two nuclei on the individual particle	**F_2_**	**111.3**	**93.5**	**113.3**
Random nucleation with three nuclei on the individual particle	**F_3_**	137.1	**116.7**	**102.6**

**Table 5:** Half-life for chitin samples extracted from *Vespa velutina* at different temperatures.

T (°C)	Time (years)
20	285
30	61
40	14
50	4
60	1
70	0.3 (110 days)
80	0.1 (37 days)
90	0.03 (12 days)
100	0.01 (5 days)
